# Bibliometric and visualization analysis of glioma pharmacotherapy from 2020 to 2024

**DOI:** 10.3389/fneur.2025.1595194

**Published:** 2025-06-13

**Authors:** Xinjie Hong, Zhentao Wei, Tian Zuo, Liyu Xu, Haohang Zhang, Tao Xu, Dongmei Wang, Chengyin Lu, Lijun Hou

**Affiliations:** ^1^Department of Neurosurgery, The Second Affiliated Hospital of Naval Medical University, Shanghai, China; ^2^School of Basic Medical Sciences, Navy Medical University, Shanghai, China

**Keywords:** glioma, pharmacotherapy, bibliometric analysis, research trends, treatment strategies

## Abstract

**Objective:**

Gliomas are the most common and aggressive malignant tumors of the central nervous system. Their complex biology and adaptive resistance mechanisms pose major obstacles to existing treatment strategies. This study aims to analyze research published over the past 5 years to identify emerging trends, effective therapeutic agents, and novel treatment strategies.

**Methods:**

Relevant articles on glioma drug therapy were retrieved from the Web of Science Core Collection for the period 2020–2024. Bibliometric analyses were performed using CiteSpace and VOSviewer to examine authorship, institutional and national contributions, journal impact, and keyword distributions. A total of 9,701 articles were included in the analysis.

**Results:**

China leads in publication volume on glioma research, followed by the United States. Liu Yang from China is the most prolific author in this field over the past 5 years. Institutions from China and the United States are the primary contributors to this area of research. Bibliometric analysis identified four major thematic clusters: glioma treatment, drug delivery, immunotherapy and the tumor microenvironment, and the molecular mechanisms underlying glioma cell behavior.

**Conclusion:**

This systematic analysis of glioma treatment research over the past 5 years highlights key global research hotspots and emerging trends. Drug delivery and immunotherapy have gained increasing attention as promising therapeutic approaches. Advances in glioma treatment are expected to improve patient outcomes.

## Introduction

Gliomas, originating from glial and neuronal cells, are the most prevalent and aggressive malignant tumors of the central nervous system (CNS) (1, 2). Glioblastoma multiforme (GBM), the most malignant subtype, accounts for 48.6% of primary malignant brain tumors. Despite advancements in treatment, GBM remains highly lethal, with a median survival of ~15 months and a 5-year survival rate of only 7.2% (3). These tumors grow diffusely, have unclear boundaries with surrounding tissue, and recur frequently, making eradication extremely difficult.

Conventional glioma treatments primarily include surgical resection, radiotherapy, and chemotherapy (4). The current standard treatment includes maximal safe resection followed by radiotherapy combined with temozolomide (TMZ) (5). However, gliomas' infiltrative growth and the blood-brain barrier (BBB) hinder treatment, leading to frequent recurrence and poor prognosis. In recent years, novel approaches such as immunotherapy and tumor-treating fields have demonstrated potential in improving patient outcomes (6, 7). Nevertheless, gliomas' complex biology and adaptive resistance remain major challenges. Current therapies often fail to fully circumvent these defense mechanisms, underscoring the urgent need for innovative treatment strategies. Developing novel therapies is essential to overcome these barriers and improve outcomes.

This study aims to identify and analyze emerging therapeutic strategies for glioma treatment. Recent advancements in glioma pharmacotherapy have opened new possibilities for overcoming treatment resistance and improving patient survival. To investigate these developments, we conducted a bibliometric analysis of glioma-related publications from 2020 to 2024, mapping research trends and predicting future hotspots in the field.

## Materials and methods

### Database and search strategy

**Figure 1:**
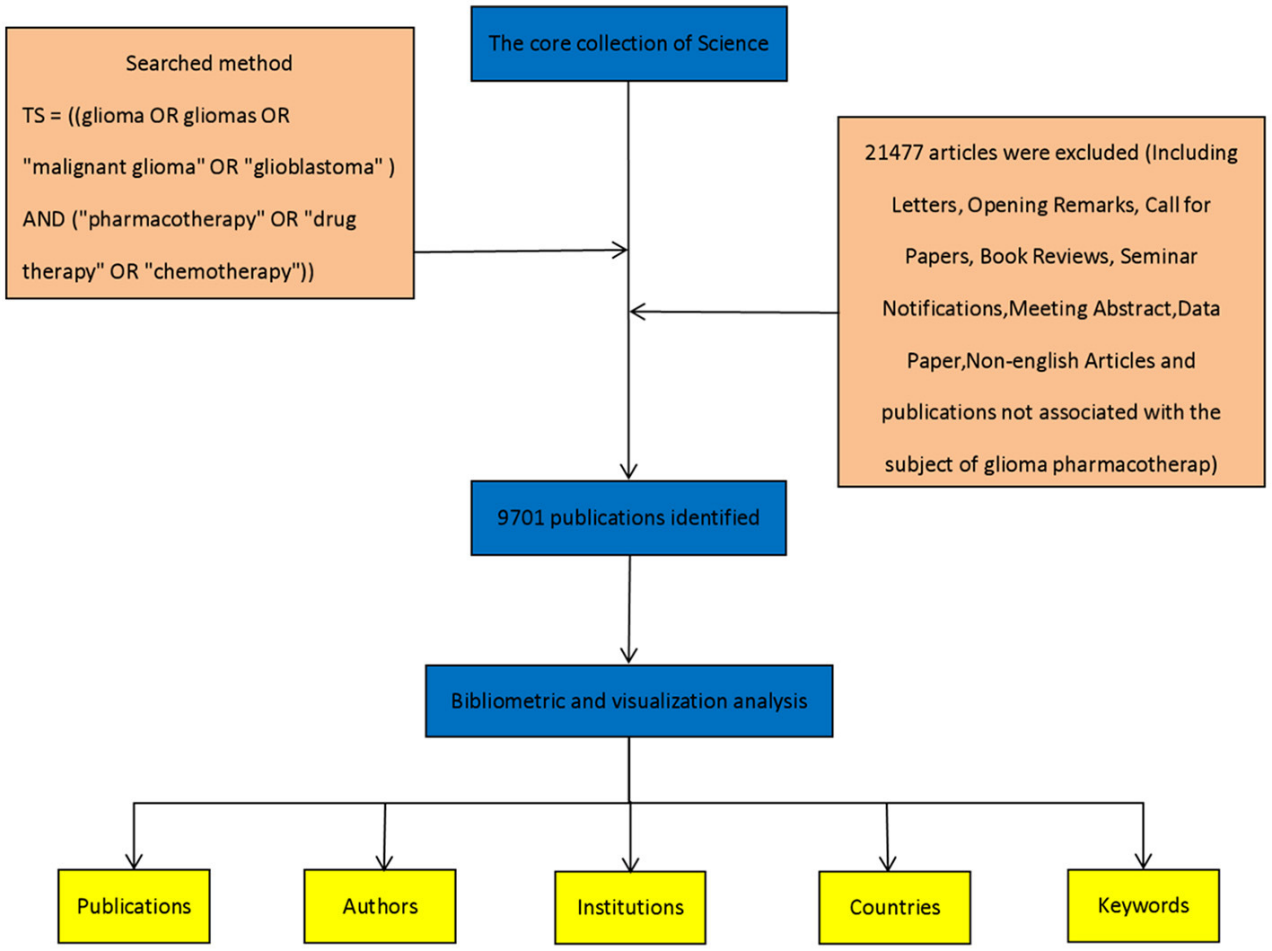
Flowchart illustrating the detailed steps of the search strategy for screening publications.

All literature data were obtained from the Web of Science Core Collection, a comprehensive database of globally influential research publications. The search was conducted on March 1, 2025, using the following query:TS = ((glioma OR gliomas OR “malignant glioma” OR “glioblastoma”) AND (“pharmacotherapy” OR “drug therapy” OR “chemotherapy”)), covering the period from January 1, 2020, to December 31, 2024. This search yielded 21,477 records. To ensure relevance and data reliability, non-academic documents-including letters, editorials, calls for papers, book reviews, and conference announcements-were excluded, resulting in a final dataset of 9,701 valid articles. The search tactics are depicted in Figure 1.

### Data analysis

We extracted bibliographic data from the Web of Science Core Collection, including titles, authors, publication years, countries/regions, institutions, keywords, citations, abstracts, and references. The data were downloaded in plain text format. VOSviewer was used to generate collaboration networks for authors, institutions, and countries/regions, as well as keyword co-occurrence and clustering maps. Citespace was applied to analyze keyword bursts and construct timeline visualizations. VOSviewer can be downloaded from https://www.vosviewer.com/, and Citespace is available at https://citespace.podia.com.

To ensure the thematic coherence and interpretability of the clustering results, we performed manual validation following automated keyword clustering. Representative keywords within each cluster were reviewed to assess their semantic consistency, and a sample of highly cited articles associated with each cluster was examined to confirm alignment with the identified themes. This process helped verify the robustness and relevance of the clustering output.

## Results

### Time distribution of publications

**Figure 2:**
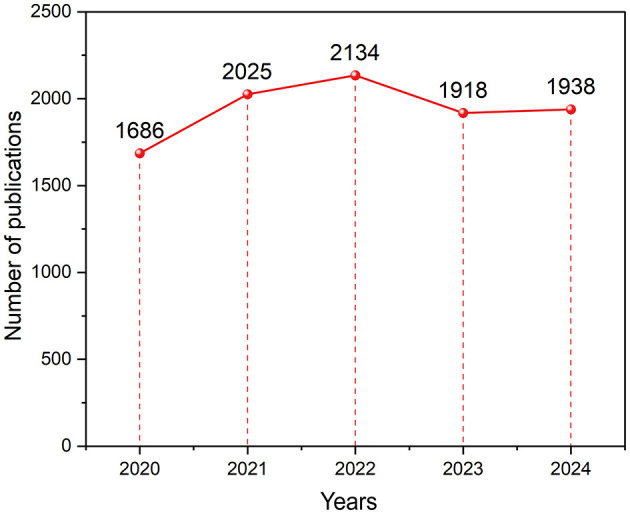
Annual trends in the number of publications in glioma pharmacotherapy from 2020 to 2024.

The annual number of publications reflects the academic development of a field (8). From 2020 to 2024, 9,701 glioma-related articles were published, as shown in Figure 2. The publication volume increased steadily from 2020 to 2022, with a significant rise in 2021 and peaking at 2,134 articles in 2022. Publication volume declined in 2023 and 2024, which may be attributed to a combination of factors, including the lingering effects of the COVID-19 pandemic, publication and indexing delays, shifting research priorities, and potential saturation of certain research topics. Nonetheless, the overall trend remained relatively stable.

### Author collaboration networks

**Figure 3:**
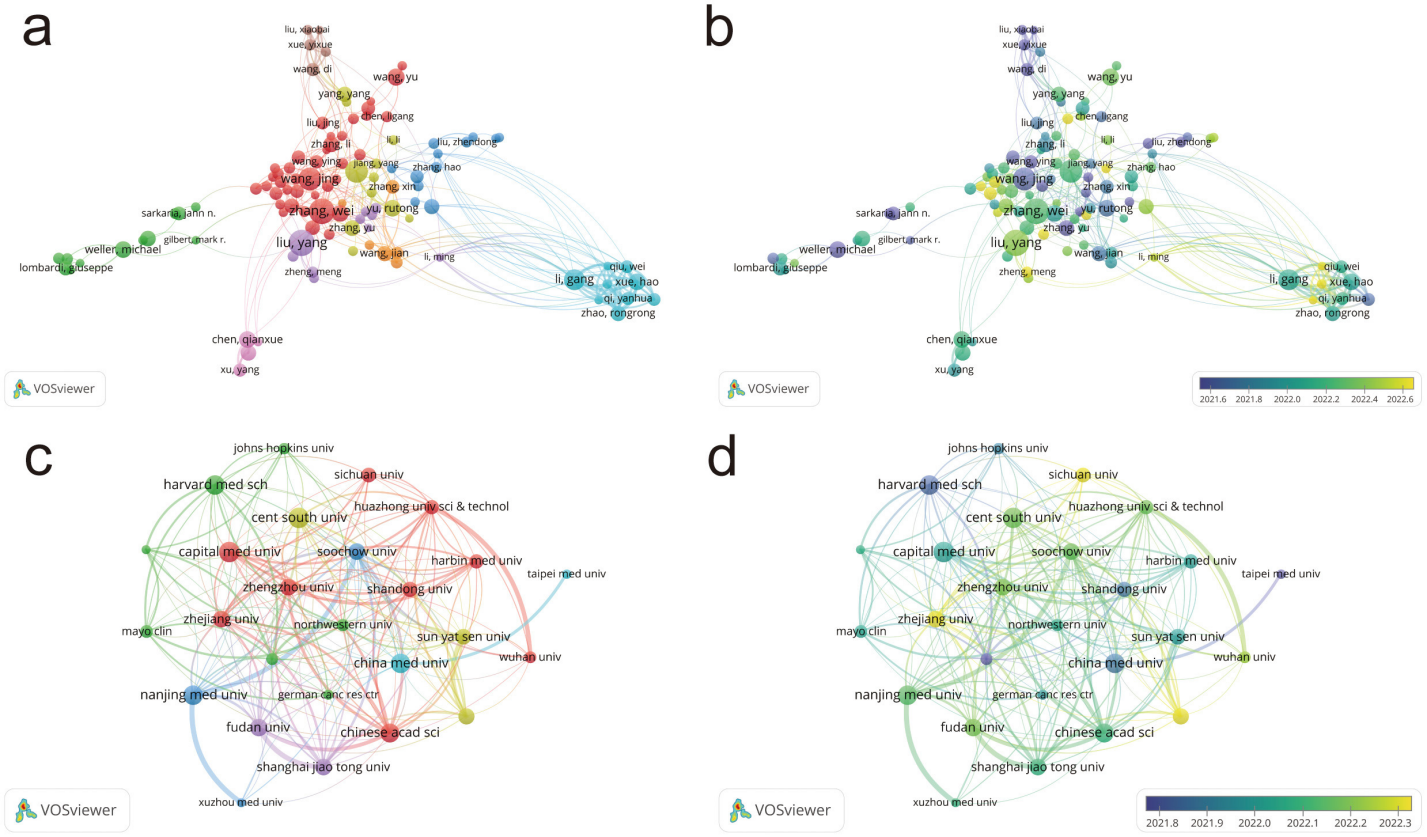
Author collaboration networks and distribution of institutions. **(a)** Author collaboration network in glioma pharmacotherapy research. **(b)** Collaboration network based on authors' average year of activity. **(c)** Distribution of institutions in glioma pharmacotherapy research. **(d)** Distribution of institutions based on average year of activity. Node color represents collaborative relationships **(a, c)** and temporal shifts in average publication year **(b, d)**; node size corresponds to frequency **(a–d)**.

Figure 3a visualizes the collaboration networks among authors in glioma research. Each node represents an author, and the connections between nodes indicate collaborative relationships. The network includes 15 authors with over 20 publications, five with over 30, and one with more than 40 publications, reflecting a high level of productivity and positive growth in the field. The top five authors are all from China, with Liu Yang (41 publications) being the most prolific, highlighting China's rapid progress in this area. The density and distribution of the connections suggest several cohesive domestic research teams with strong interactions, while international research appears more fragmented, though there is some collaboration between domestic and international teams.

Glioma research has a long-standing history, with scholars from various countries contributing extensively to the establishment of a robust academic foundation. In recent years, a growing number of emerging researchers from China have produced a high volume of publications, positioning the country as a leading force in the field and underscoring its rapid progress in glioma research (Figure 3b).

### Distribution of institutions

Using VOSviewer, we visualized the key institutions and their collaboration networks, as shown in Figure 3c. The analysis reveals that higher education institutions and research organizations dominate the field, reflecting their central role in glioma research. Chinese institutions, particularly Shandong University, Nanjing Medical University, and Capital Medical University, play a leading role. These institutions exhibit strong, balanced collaboration, creating a dynamic and thriving research environment.

Figure 3d illustrates that glioma research abroad began earlier, with institutions like Harvard Medical School and Johns Hopkins University leading the way. In China, early contributors include Shandong University, China Medical University, and National Taiwan University. In recent years, numerous domestic institutions have achieved considerable advancements, contributing to the development of a comprehensive and multi-layered research network in the field.

### Country and region distribution

**Figure 4:**
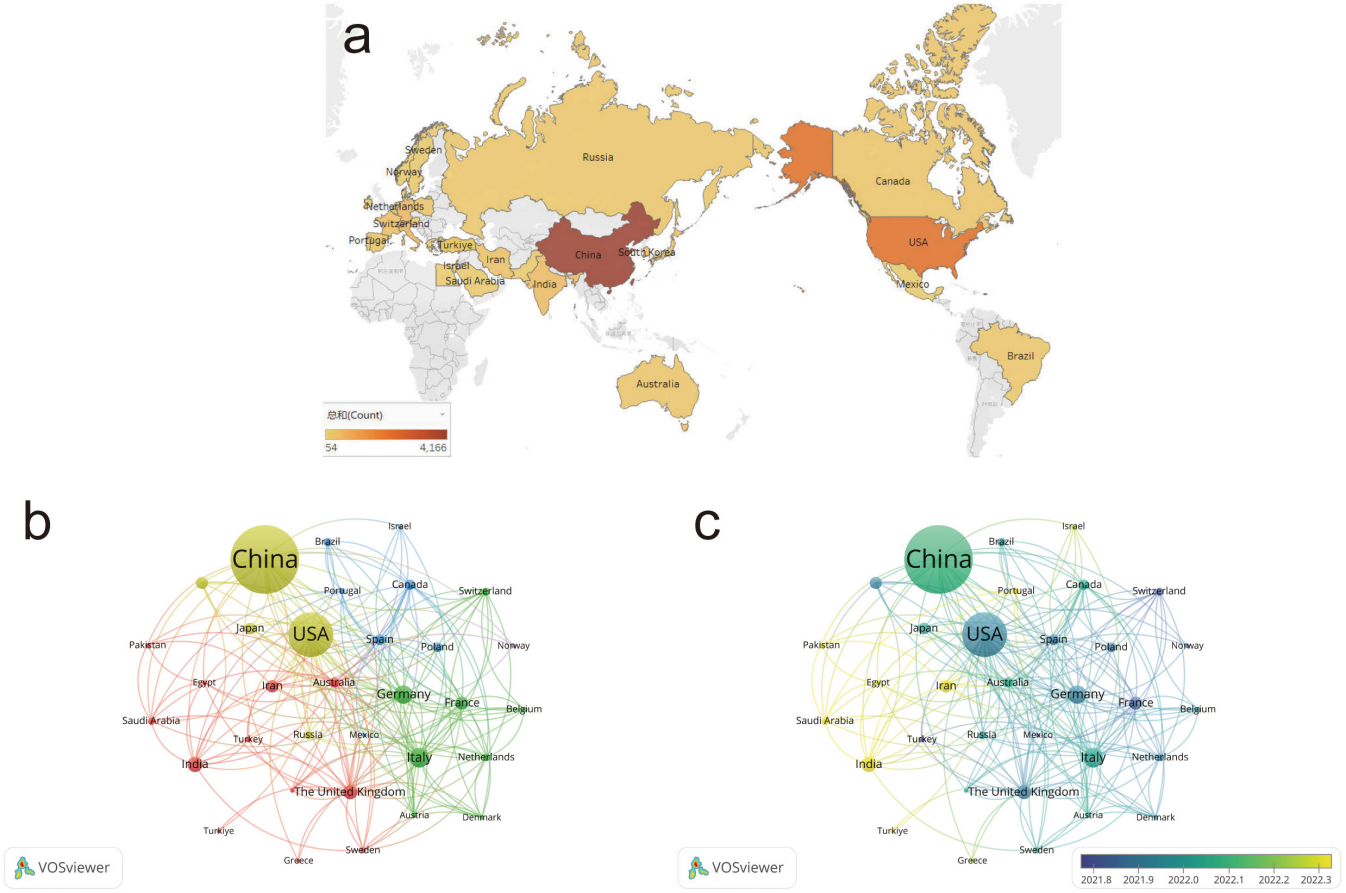
Country and region distribution. **(a)** Geographic distribution of published articles in glioma research. **(b)** Country distribution and collaboration network in glioma research. **(c)** Country distribution and collaboration network based on average year of activity. Node color represents collaborative relationships **(b)** and temporal shifts in average publication year **(c)**; node size corresponds to frequency **(b, c)**.

Figure 4a shows the distribution of publications in glioma research across countries. China ranks first globally in publication output, with the United States following closely behind. Research is primarily concentrated in developed countries in Europe and North America. Using VOSviewer, we analyzed the collaborative relationships among major countries and regions (Figure 4b). Figure 4c reveals that developed countries, led by the United States, began their research in this field earlier. China has experienced rapid growth in recent years. In contrast, some developing countries in Asia and Africa have only recently started to emerge in this area.

### Keyword co-occurrence network analysis

**Figure 5:**
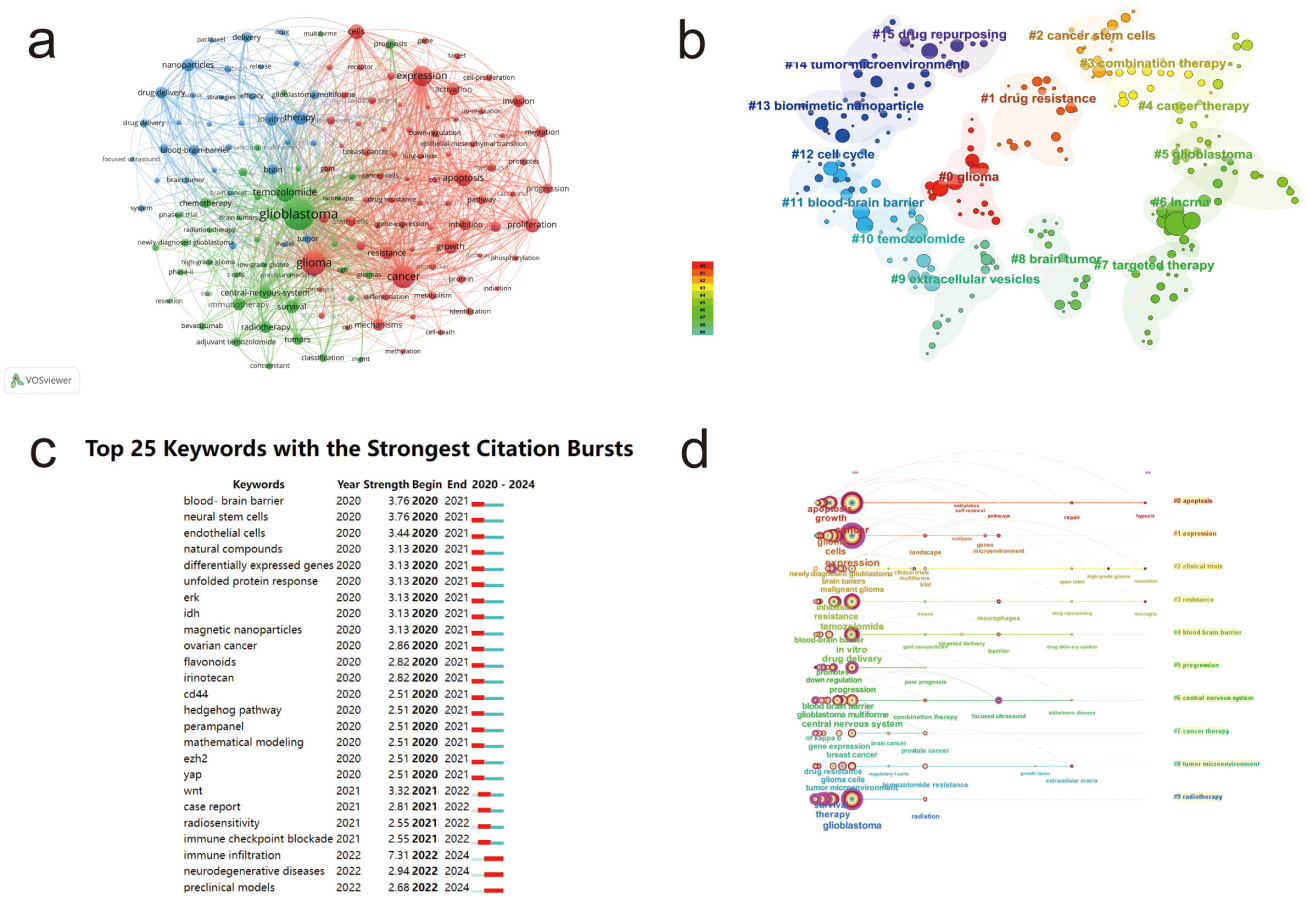
Keyword analysis in glioma research. **(a)** Co-occurrence network of keywords in glioma research. **(b)** Clustering map of keywords in glioma research. **(c)** Burst detection map of keywords in glioma research. **(d)** Timeline map of keywords in glioma research. Node color represents clustering relationships **(a, b)** and node size corresponds to frequency **(a, b)**.

Keywords are concise summaries of the main themes in literature. Analyzing keywords can reveal research trends, hotspots, and connections between themes in a field. In this study, we used Citespace and VOSviewer to analyze keywords (Figure 5a). The results show that terms such as “glioblastoma,” “glioma,” “expression,” “apoptosis,” “tumor,” and “temozolomide” are highly frequent keywords. These keywords form a complex network with extensive radiating connections to other terms like “nanoparticles,” “blood-brain barrier,” “immunotherapy,” “central nervous system,” “proliferation,” and “treatment.” This indicates that the field of glioma research has developed a well-defined content system.

### Keyword clustering analysis

Based on the keyword co-occurrence network, we clustered keywords with similar characteristics to create a clustering map in the field of glioma research (Figure 5b). The keywords were divided into 15 clusters: #0 glioma, #1 drug resistance, #2 cancer stem cells, #3 combination therapy, #4 tumor treatment, #5 glioblastoma, #6 lncRNA, #7 targeted therapy, #8 brain tumors, #9 extracellular vesicles, #10 temozolomide, #11 blood-brain barrier, #12 cell cycle, #13 biomimetic nanoparticles, #14 tumor microenvironment, and #15 drug repurposing. By merging similar cluster terms and integrating high-frequency keywords, we identified two main research directions in glioma studies: immunotherapy and tumor microenvironment, and drug delivery for glioma treatment.

### Keyword burst and time-zone analysis

Keyword burst and time-zone analyses (Figures 5c, d) reveal the historical evolution of glioma research by identifying emerging trends and the longevity of research hotspots. In 2020, 18 new keywords emerged, including “blood-brain barrier,” “neural stem cells,” and “endothelial cells.” In 2021, additional keywords such as “Wnt signaling protein,” “case report,” “immune checkpoint inhibition,” and “radiosensitivity” appeared. By 2022, terms such as “immune infiltration,” “preclinical models,” and “neurodegenerative diseases” began to gain prominence and have maintained their relevance through 2024, reflecting sustained and growing research interest in these areas. The year 2020 marked a peak in keyword emergence, with 18 new terms introduced. Among these, “immune infiltration” had the highest burst strength (7.31), underscoring its prominence as a major research hotspot in the field.

## Discussion

### Global research status and trends

Bibliometric analysis offers a comprehensive overview of research trends in glioma pharmacotherapy. China and the United States are the leading contributors in this field, with both countries generating a substantial volume of high-quality research. This prominence reflects their robust research infrastructures and considerable investments in the field of neuro-oncology. Notably, collaborations between Chinese and U.S. scientists have contributed to important progress in glioma research, including studies on brain metastases at the single-cell level (9) and neural circuit interactions in glioblastoma (10). These efforts have enhanced our understanding of tumor biology and highlight how international cooperation can drive innovation and high-impact discoveries in the field. Additionally, countries in Europe, Asia, and the Americas contribute to the globalization of glioma research, each focusing on specific areas. For example, Europe has become a leader in innovative immunotherapy research (11, 12). Although China and the United States dominate in publication output, there is a modest but noticeable increase in contributions from developing regions. Continued growth may be supported by enhanced international collaboration, targeted funding, and improved access to research infrastructure and publishing platforms.

Keyword analysis reveals major focus areas in glioma pharmacotherapy. High-frequency terms like “glioblastoma,” “temozolomide,” and “immunotherapy” form a strong research network, indicating their central role in current studies. The frequent appearance of terms like “immune infiltration” and “immunotherapy” shows the rising prominence of immune-based approaches, reflecting substantial advances in tumor immunology (13, 14). Additionally, the continued emphasis on keywords such as “blood-brain barrier” and “drug delivery” underscores the ongoing challenge of overcoming the blood-brain barrier to enhance drug efficacy (15, 16).

Regarding research trends, 2020 marked a surge in keyword emergence, signaling the rapid expansion of glioma research. Recent keywords like “neurodegenerative disease” and “preclinical modeling” suggest that research is increasingly shifting toward understanding basic mechanisms and advancing translational medicine (17, 18). This trend aligns with neuro-oncology's aim to develop better therapies through deeper insights into tumor biology.

### Pharmacotherapy and immunotherapy

Chemotherapy, particularly TMZ, has been the standard treatment for glioblastoma for many years. TMZ induces DNA damage, causing cell cycle arrest and apoptosis in tumor cells (19). However, resistance to TMZ is common, driven by mechanisms such as MGMT promoter methylation and other genetic alterations (20). To overcome these challenges, combination therapies integrating TMZ with agents like radiation therapy or immunotherapy are being explored (21, 22). Immunotherapy has emerged as a promising approach in glioma treatment. Immune checkpoint inhibitors, such as anti-PD-1 and anti-CTLA-4 antibodies, enhance the immune system's ability to target tumor cells (23, 24). However, the response to these therapies is often limited by the immunosuppressive tumor microenvironment (TME). Recent studies indicate that combining immune checkpoint inhibitors with treatments like radiation therapy or CAR-T cell therapy can improve therapeutic outcomes (25, 26).

Combining pharmacotherapy with immunotherapy has shown promise in both preclinical and clinical studies. For instance, a phase 2 trial combining TMZ with pembrolizumab (an anti-PD-1 antibody) and TTFields demonstrated improved progression-free survival in newly diagnosed glioblastoma patients (27). Additionally, oncolytic viruses, which selectively infect and destroy tumor cells while stimulating an immune response, have shown synergistic effects when combined with immune checkpoint inhibitors (28–30). Future research should focus on optimizing combination therapies to maximize efficacy and minimize toxicity. Exploring new targets within the TME, such as the cGAS-STING pathway, could provide potential for reprogramming the immune response. Moreover, addressing the challenges of the BBB is crucial, as effective drug delivery to the brain is essential for successful treatment. The emergence of keywords such as “immune infiltration” underscores the increasing attention to the tumor microenvironment in glioma, particularly the role of immune cells in modulating tumor progression and response to immunotherapies.

The keyword “immune infiltration” showed the strongest burst strength in our bibliometric analysis, highlighting the growing recognition of immune cells-including tumor-associated macrophages, T cells, and microglia-as key regulators of glioma progression and treatment responsiveness. This trend reflects a paradigm shift toward therapeutic strategies that not only target tumor cells directly but also modulate immune dynamics within the TME to enhance the success of immunotherapy. Although immunotherapy has attracted increasing research interest, its clinical application in glioma remains limited, with most approaches still being evaluated in early-phase trials or exploratory combination regimens (31, 32).

### Pharmacotherapy and drug delivery

The BBB is a major obstacle in glioma treatment, as its selective permeability limits the entry of therapeutic agents into the brain. Extensive research has focused on strategies to enhance drug delivery across the BBB and increase its permeability. These strategies can be categorized into physical, chemical, and biological methods. Physical approaches, such as focused ultrasound (33), andmicrobubble-assisted delivery (34), transiently disrupt the BBB, increasing its permeability without causing permanent damage. Chemical methods, including receptor-mediated transcytosis (RMT), utilize the natural transport mechanisms of the BBB to facilitate drug delivery. Biological approaches involve functionalizing nanocarriers with ligands targeting specific receptors on endothelial cells, such as transferrin receptor (35), to improve drug delivery efficiency through RMT.

Nanotechnology has emerged as a promising solution for overcoming the BBB in glioma treatment. Among the emerging nanoparticle platforms, exosomes, polymeric micelles, and biomimetic nanocarriers have gained increasing prominence in recent glioma research (36, 37). Exosomes, as naturally occurring extracellular vesicles, offer intrinsic advantages such as high biocompatibility, low immunogenicity, and innate ability to cross the BBB. Polymeric micelles, with their core-shell architecture, are particularly useful for encapsulating hydrophobic drugs and enabling sustained release. In addition, biomimetic nanoparticles—engineered by coating synthetic carriers with cell membranes [e.g., leukocytes (38), platelets (39), or glioma cells (16)]—enhance immune evasion, prolong circulation time, and improve targeting specificity. These advanced delivery systems represent a promising frontier in overcoming the physiological barriers to effective glioma treatment and are increasingly highlighted.

The frequent appearance of keywords such as “nanoparticles” in recent literature reflects the growing interest in nanotechnology-based delivery systems, which offer a viable strategy to bypass the BBB and deliver chemotherapeutic or immunomodulatory agents more effectively to glioma tissues. This aligns with ongoing efforts to improve drug bioavailability and therapeutic outcomes through innovative formulation techniques. Notably, several early-phase clinical trials—such as NCT03566199, NCT04881032, and NCT03020017—have been initiated to evaluate the clinical potential of nanoparticle-based approaches for glioma therapy. However, most remain in Phase I/II, indicating that while preclinical data are promising, clinical translation is still in its early stages. These observations suggest that high-frequency keywords like “nanoparticles” reflect emerging innovation rather than widespread clinical adoption, underscoring both the promise and the ongoing challenges of translating nanotechnology into approved therapies.

## Conclusion

This study offers a comprehensive analysis of glioma drug therapy research published over the past 5 years, identifying key global research trends. Drug delivery and immunotherapy are highlighted as primary areas of focus, reflecting a growing interest in innovative strategies to address glioma treatment challenges. The findings suggest that continued advancements in these fields hold promising potential to overcome current treatment limitations.

## Limitations

Despite the comprehensive nature of this bibliometric analysis, several limitations should be noted. First, it relies solely on the Web of Science Core Collection, which may exclude relevant studies indexed in other databases, potentially introducing selection bias. Second, only English-language publications were analyzed, possibly overlooking important research published in other languages. Third, while the study highlights research trends, it does not evaluate the clinical impact of these therapies. Finally, due to indexing delays, literature from late 2024 may be incomplete, leading to a slight underestimation of publications for that year.

## Data Availability

The original contributions presented in the study are included in the article/supplementary material, further inquiries can be directed to the corresponding authors.
